# Mannose-Binding Lectin 2 (MBL2) combined genotypes deficiency is associated with susceptibility for Oral Lichen Planus

**DOI:** 10.1590/1678-4685-GMB-2018-0015

**Published:** 2019-02-21

**Authors:** Vania Polesello, Ludovica Segat, Matteo Biasotto, Giulia Ottaviani, Margherita Gobbo, Roberto Di Lenarda, Sergio Crovella, Luisa Zupin

**Affiliations:** 1 Institute for Maternal and Child Health Institute for Maternal and Child Health IRCCS Trieste Italy Institute for Maternal and Child Health, IRCCS “Burlo Garofolo”, Trieste, Italy; 2 Division of Oral Medicine and Pathology Division of Oral Medicine and Pathology Trieste Italy Division of Oral Medicine and Pathology, Dental Clinic, “Maggiore” Hospital, Trieste, Italy; 3 University of Trieste University of Trieste Department of Surgical, Medical and Health Sciences Trieste Italy Department of Surgical, Medical and Health Sciences, University of Trieste, Trieste, Italy

**Keywords:** Mannose-Binding Protein-C, MBL2, Oral Lichen Planus, polymorphisms

## Abstract

Oral Lichen Planus (OLP) is an oral inflammatory condition, mediated by host immune system reaction, presenting basal membrane damages with inflammatory lesions in the mouth and/or skin. In this study, the role of functional polymorphisms in the MBL2 gene, encoding for Mannose-Binding Protein C (MBP-C), a member of the innate immune response and an acute-phase protein able to activate the complement cascade, was investigated to assess a possible association with OLP susceptibility in Italian patients. Two variations at the promoter region (called H/L and X/Y) and three at the first exon (at codon 52, 54, and 57) of the MBL2 gene were analyzed in 69 OLP patients and 244 healthy controls from northeastern Italy. Considering the polymorphisms singularly, the MBL2 X allele and C/T genotype of the D allele (correlated with low MBP-C expression) were associated with susceptibility to develop OLP. Moreover, when taking into account MBL2 combined genotypes, more OLP patients were deficient MBP-C producers than not deficient, who were more represented among healthy controls. MBL2 combined genotypes, responsible for deficient MBP-C production, are associated with an increased risk of developing OLP.

## Introduction

Oral Lichen Planus (OLP) is a chronic inflammatory disease with unknown etiology that affects the oral mucosa (Wang and van der Waal, 2015). The current hypothesis about OLP etiology and pathogenesis proposes that a T-cell mediated process causes tissue lesions, increasing local cytokines production and altering the expression of adhesion molecules (De Rossi and Ciarrocca, 2014). OLP pathogenesis involves a series of complex non-specific antigens as well as specific mechanisms, and considering the latter, it has been suggested that basal keratinocyte apoptosis is triggered by CD4- and CD8-activated lymphocytes and inflammatory cytokines (Sugerman *et al.*, 2002; Roopashree *et al.*, 2010).

OLP is characterized by damaged basal membrane areas forming colloid bodies, resulting in inflammatory eruptive lesions with reddish-purple spots in the mouth and/or skin. More aggressive forms present atrophy and/or erosion accompanied by pain and burning sensation (Mollaoglu, 2000; de Moura Castro Jacques *et al.*, 2003; Lukac *et al.*, 2006). OLP affects predominantly adults over 40 years old and occurs more in women than in men (ratio ~ 3:2) (Sugerman *et al.*, 2002; Arora *et al.*, 2016; Mozaffari *et al.*, 2016). At present, a cure for OLP is not available and the only treatment is for symptoms relief (De Rossi and Ciarrocca, 2014).

The Mannose-Binding Protein C (MBP-C, also known as, Mannose-Binding Lectin MBL) is a serum acute-phase glycoprotein produced by the liver with a key role in innate immunity (Turner, 2003). MBP-C is able to activate the lectin pathway of the complement cascade through the binding of mannose residues present on the surface of pathogens (Ikeda *et al.*, 1987), via the MBP-C-associated serine protease (MASP)-2 pathway (Thiel *et al.*, 1997).

MBP-C production is enhanced by inflammatory stimuli (Arai *et al.*, 1993), but it is also genetically determined. The *MBL2* gene (10q11.2-q21) encodes the MBP-C protein (Madsen *et al.*, 1994), and two polymorphisms at position -550 (called H/L, rs11003125) and -221 (called X/Y rs7096206) located at the promoter region have been reported to be associated with lower MBP-C serum concentration (Garred *et al.*, 1995). Three single-base substitutions in the *MBL2* exon-1 at codon 52 (allele D, C >T, rs5030737), codon 54 (allele B, G >A, rs1800450), and codon 57 (allele C, G >A, rs1800451) have been named allele O (Sumiya *et al.*, 1991; Madsen *et al.*, 1994), since these polymorphisms have the same detrimental effect on MBP-C serum concentration, impairing oligomerization and leading to functional deficiency; the wild type allele was named allele A (Garred *et al.*, 1995; Wallis and Cheng, 1999). Taken together, *MBL2* promoter and exon 1 polymorphisms combine to form six different haplotypes (HYA, LYA, LXA, HYD, LYB, LYC) that are correlated with different MBP-C serum concentrations (Turner, 2003; Garred, 2008).

Considering that OLP is an immunologically mediated disease, it could be possible that a low MBP-C serum concentration, negatively influencing the host innate immune response, may impact OLP pathogenesis. To date, just one study investigated *MBL2* polymorphisms in OLP patients, without evidencing any correlation between *MBL2* A/O variants and OLP susceptibility in Brazilian patients (Barkokebas *et al.*, 2011). Aiming to disclose the possible role of *MBL2* functional genetic variants in the susceptibility for OLP, we analyzed *MBL2* promoter and exon 1 polymorphisms in a group of OLP patients and healthy controls from northeastern Italy.

## Subjects and Methods

### Patient population

A total of 69 Italian OLP patients (51 women and 18 men, median age = 65 years, range = 37-89) and 244 healthy Caucasian individuals (188 women and 56 men, median age = 55 years, range = 39-73) were enrolled at the Division of Oral Medicine and Pathology (Dental Clinic, University of Trieste, Trieste, Italy). A blood sample was collected from each patient, immediately stored at 4°C for no more than 24 hours, and transported at the Institute for Maternal and Child Health IRCCS “Burlo Garofolo”, Trieste, Italy.

Patients considered with clinical suspicion of OLP had bilateral white reticulations in either the buccal mucosa or tongue, regardless of whether erythema and ulcers were present. Patients with bilaterally symmetric erythema of the gingiva with or without striations were also enrolled. Patients with unilateral lesions or lichenoid reactions, with concomitant autoimmune disorders or undergoing immunomodulating pharmacological therapies were excluded from the study. Histopathologic confirmation was obtained for each patient after biopsy performed with local anesthesia. We then considered the clinical characteristics of the OLP patients: more than 80% were affected by typical bilateral hyperkeratotic reticulated white striae in the buccal mucosa. A third of OLP patients were affected by upper and lower desquamative gingivitis, sometimes recorded as an isolated feature (10%). Ulcerative OLP was less frequent, with the highest percentage found on bilateral buccal mucosa and tongue. Palate and floor of the mouth were the most spared sites.

In females, 15.2% reported genital involvement, 21.2% cutaneous involvement, and 6.2% had involvement of both sites. In males, 5.9% had involvement of both sites, whereas none of the men had an isolated genital or cutaneous involvement.

Informed written consent was obtained from all patients and healthy controls. This work was in accordance with the ethical standards of the 1975 Declaration of Helsinki (7^th^ revision, 2013) and the Bio-Ethical Committee of IRCCS Burlo Garofolo approved the study (RC03/04, L1055, protocol number 118/10).

### MBL2 genotyping

Genomic DNA was extracted from peripheral whole blood using NucliSENS^®^ easyMAG^®^ (BioMérieux, Marcy l’Etoile, France) automated system for total nucleic acid extraction. *MBL2* genotyping was performed using the following primers: for promoter polymorphisms, forward 5’ CCA GGG CCA ACG TAG TAA GA 3’ and reverse 5’ GAG GGG TTC ATC TGT GCC TA 3’, for exon 1 polymorphisms, forward 5’ GGG CAT GCT CGG TAA ATA TG 3’ and reverse 5’ TGC CCA GAG AAT GAG AGC TGA 3’. Polymerase chain reactions (PCR) were carried out in a Gene-Amp 9700 Thermal cycler (Applied Biosystems – Thermo Fisher Scientific, Foster City, California, U.S.A.) using PCR buffer 1x, 1 unit of Taq Gold enzyme, 0.2 mM dNTPs, and 2 mM MgCl_2_. The PCR protocol was as follows: denaturation for 30 s at 95 °C; annealing for 30 s at 55 °C; extension for 30 s at 72 °C, for 40 cycles. PCR products were visualized after electrophoresis in a 2% agarose gel to check successful DNA amplifications and the absence of non-specific reaction products. DNA sequencing was performed using BigDye Terminator Cycle Sequencing Ready Reaction Kit 2.0 (Applied Biosystem) on an automated ABI Prism 3130 Genetic Analyser, PE, using the 3130 Data Collection Software (Applied Biosystem). Sequences were analyzed using the 4Peaks software (http://nucleobytes.com/index.php/4peaks). Allele and genotype frequencies of *MBL2* gene polymorphisms were calculated by direct counting.

We also analyzed promoter and exon 1 haplotypes, as well as combined genotypes. Using common nomenclature, the first two letters indicate the promoter region variants (alleles H/L and X/Y) and the third letter indicates the combination of the three polymorphisms in exon 1 (A/O allele). The combined genotypes were classified according to Bouwman *et al.* (2006) as high (HYA/HYA, HYA/LYA, LYA/LYA, HYA/LXA, LYA/LXA), low (LXA/LXA, HYA/O, LYA/O) and deficient (LXA/O, O/O) producers.

### Statistical analysis

Fisher’s exact test was used for pairwise comparison of alleles, genotypes, and combined genotypes frequencies using contingency tables as appropriate and *p*-values <0.05 were considered significant. All statistical analyses were carried out using the open-source R software version 3.1.0 (R CoreTeam, 2015). Post hoc power calculations were performed through G*Power software version 3.1.9.2 (Faul *et al.*, 2007).

## Results

All *MBL2* SNPs analyzed were in Hardy-Weinberg (HW) equilibrium, with the exception of the C 57 G >A polymorphism in the OLP group (Table 1). *MBL2* polymorphisms frequency distribution in our healthy subjects was similar to those reported by the 1000 Genomes Project for the European Tuscan population (Table 2) (1000 Genomes Project Consortium *et al.*, 2012).

**Table 1:** *MBL2* polymorphisms allele, genotype and haplotype counts (and frequency) in OLP patients (OLP) and healthy subjects (HC).

*MBL2*	OLP n=69	HC n=244	OLP vs. HC *p*-value; Odds Ratio; 95% Confidence Interval
H/L rs11003125			
L	90 (0.65)	309 (0.63)	Ref.
H	48 (0.35)	121 (0.27)	*p*=0.16; OR=1.36; 95%CI=0.88-2.08
L/L	31 (0.45)	101 (0.41)	Ref.
H/L	28 (0.41)	107 (0.44)	*p*=0.66; OR=0.85; 95%CI=0.46-1.58
H/H	10 (0.14)	36 (0.15)	*p*=1.00; OR=0.90; 95%CI=0.36-2.14
*HW*	χ^2^=0.45; *p*=0.50	χ^2^=0.76; *p*=0.38	
X/Y rs7096206			
Y	101 (0.73)	396 (0.81)	Ref.
X	37 (0.27)	92 (0.19)	*p*=0.04; OR=1.58; 95%CI=0.98-2.49
Y/Y	39 (0.57)	162 (0.66)	Ref.
X/Y	23 (0.33)	72 (0.29)	*p*=0.36; OR=1.32; 95%CI=0.70-2.47
X/X	7 (0.10)	10 (0.04)	*p*=0.06; OR=2.89; 95%CI=0.87-9.04
*HW*	χ^2^=1.01; *p*=0.31	χ^2^=0.31; *p*=0.58	
D rs5030737			
C	124 (0.90)	456 (0.93)	Ref.
T	14 (0.10)	32 (0.07)	*p*=0.19; OR=1.61; 95%CI=0.77-3.21
C/C	55 (0.80)	228 (0.94)	Ref.
C/T	14 (0.20)	16 (0.06)	*p*=0.002; OR=3.61; 95%CI=1.53-8.43
T/T	0 (0.00)	0 (0.00)	
*HW*	χ^2^=0.13; *p*=0.72	χ^2^=0.28; *p*=0.60	
B rs1800450			
G	122 (0.88)	409 (0.84)	Ref.
A	16 (0.12)	79 (0.16)	*p*=0.24; OR=0.68; 95%CI=0.36-1.23
G/G	55 (0.80)	171 (0.70)	Ref.
G/A	12 (0.17)	67 (0.27)	*p*=0.11; OR=0.56; 95%CI=0.25-1.14
A/A	2 (0.03)	6 (0.02)	*p*=1.00; OR=1.04; 95%CI=0.10-6.01
*HW*	χ^2^=0.55; *p*=0.46	χ^2^=0.03; *p*=0.85	
C rs1800451			
G	136 (0.99)	484 (0.99)	Ref.
A	2 (0.01)	14 (0.01)	*p*=0.54; OR=0.51; 95%CI=0.05-2.26
G/G	67 (0.97)	230 (0.96)	Ref.
G/A	2 (0.03)	14 (0.06)	*p*=0.54; OR=0.49; 95%CI=0.05-2.22
A/A	0 (0.00)	0 (0.00)	
*HW*	χ^2^=16.38; *p*=5.18e-05	χ^2^=0.21; *p*=0.64	
A/O			
A	105 (0.76)	379 (0.78)	Ref.
O	33 (0.24)	109 (0.22)	*p*=0.73; OR=1.09; 95%CI=0.68-1.73
A/A	40 (0.58)	148 (0.61)	Ref.
A/O	25 (0.36)	83 (0.34)	*p*=0.77; OR=1.11; 95%CI=0.60-2.03
O/O	4 (0.06)	13 (0.05)	*p*=0.76; OR=1.14; 95%CI=0.25-3.95
*HW*	χ^2^=0.06; *p*=0.80	χ^2^=0.09; *p*=0.76	
MBP-C producers			
MBP-C High Producers	31 (0.45)	140 (0.58)	Ref.
MBP-C Low Producers	24 (0.35)	91 (0.37)	*p*=0.65; OR=1.19; 95%CI=0.62-2.24
MBP-C Deficient Producers	14 (0.20)	13 (0.05)	*p*=0.0003; OR=4.81; 95%CI=1.89-12.39

**Table 2:** *MBL2* allele and genotype frequencies in the present study and Tuscan population.

*MBL2*	HC (current study)	TSI 1000 GP^a^	*MBL2*	HC (current study)	TSI 1000 GP^a^	*MBL2*	HC (current study)	TSI 1000 GP^a^
	n=244	n=107		n=244	n=107		n=244	n=107
H/L rs11003125			X/Y rs7096206					
L	309 (0.63)	136 (0.63)	Y	396 (0.81)	172 (0.80)			
H	121 (0.27)	78 (0.36)	X	92 (0.19)	42 (0.20)			
L/L	101 (0.41)	43 (0.40)	Y/Y	162 (0.66)	68 (0.64)			
H/L	107 (0.44)	50 (0.47)	X/Y	72 (0.29)	36 (0.34)			
H/H	36 (0.15)	14 (0.13)	X/X	10 (0.04)	3 (0.03)			
D rs5030737			B rs1800450			C rs1800451		
C	456 (0.93)	205 (0.96)	G	409 (0.84)	182 (0.85)	G	484 (0.99)	214 (1.00)
T	32 (0.07)	9 (0.04)	A	79 (0.16)	32 (0.15)	A	14 (0.01)	0 (0.00)
C/C	228 (0.94)	98 (0.92)	G/G	171 (0.70)	79 (0.74)	G/G	230 (0.96)	107 (1.00)
C/T	16 (0.06)	9 (0.08)	G/A	67 (0.27)	24 (0.22)	G/A	14 (0.06)	0 (0.00)
T/T	0 (0.00)	0	A/A	6 (0.02)	4 (0.04)	A/A	0 (0.00)	0 (0.00)

a 1000 GP = 1000 Genomes Project Consortium *et al.* (2012)

The comparison of *MBL2* allelic and genotypic frequency between OLP patients and healthy controls is reported in Table 1. Five common *MBL2* haplotypes were identified: HYA, LYA, LXA, HYO, LYO. They were further arranged in combined genotypes and assembled according to the corresponding effect on MBP-C production (Table 1).

Taking into account the polymorphisms singularly, the X allele was more frequent in OLP patients compared to healthy controls (*p*=0.04; OR=1.58; 95%CI=0.98-2.49) (Table 1). Moreover, C/T genotype of exon 1 D polymorphism was more frequent among OLP patients than the homozygous C/C genotype, which was more frequent among healthy controls (*p*=0.002; OR=3.61; 95%CI=1.53-8.43) (Table 1). No significant difference in frequency distribution was found between OLP patients and healthy controls for the other polymorphisms (Table 1).

When considering *MBL2* combined genotype, the deficient producer (DP) combined genotype was more frequent among OLP patients than the high producer (HP) one, more frequent among healthy controls (*p*=0.0003; OR=4.81; 95%CI=1.89-12.39) (Table 1). Finally, when stratifying OLP patients according to their clinical manifestations, no association was found with specific *MBL2* genotypes, haplotypes, or combined genotypes.

The power analysis showed a low power for X/Y alleles using Fishers post hoc test (power=0.50, α=0.04), a quite high power for D allele genotypes (power=0.83, α=0.03), a high power for combined genotypes using a χ^2^ post hoc test (power=0.99, non-centrality parameter λ=47.32, critical χ2=5.99) and Fisher’s post hoc test (DP combined genotype: power=0.93, actual α=0.03).

## Discussion

OLP is a multifactorial disease with several genes and environmental factors involved in its etiopathogenesis (Sugerman *et al.*, 2002), which is not yet completely understood. To date, several studies analyzed the genetic variants involved in OLP susceptibility. Most recently, the *IL-17A* G197A polymorphism has been reported to be involved in the susceptibility to OLP in Brazilian patients (Gueiros *et al.*, 2017). Moreover, the *IFNG* (874A/T) polymorphism has also been associated with OLP in a Saudi Arabian population (Al-Mohaya *et al.*, 2016). In an Egyptian population, the presence of *IL18*-137 GG and *IL10*-592 AA genotypes, as well as *IL18*-137G and *IL10*-592A alleles, have been correlated with OLP (Abdel Hay *et al.*, 2016). Finally, *VDR* FokI and *CYP24A1* gene polymorphisms have been also observed to be linked to OLP in patients and controls from Serbia (Kujundzic *et al.*, 2016).

To our knowledge, only one study has previously investigated the role of *MBL2* A/O alleles in OLP, yet without finding a correlation with susceptibility to the disease (Barkokebas *et al.*, 2011), as in our study. When we evaluated *MBL2* polymorphisms singularly, the X allele and C/T genotype of the D allele associated with low MBP-C expression (Bouwman *et al.,* 2006), were associated with increased risk of OLP development, as well as the *MBL2* combined genotypes, and the fact that DP was more frequent in OLP patients than HP, which is more common among healthy controls, suggests a possible involvement of *MBL2* in susceptibility to OLP development. Barkokebas *et al.* (2011) did not analyze promoter polymorphisms and consequently haplotypes and combined genotypes, so a comparison with our results was not possible.

The finding that individuals carrying *MBL2* DP genotypes are more susceptible to OLP leads us to hypothesize a scenario in which low MBP-C levels are responsible for excessive cytokines (TNF-α, IL-1b and IL-6) activation, impacting on the basal keratinocyte apoptosis thus negatively influencing the development of the disease (Dean *et al.*, 2011).

Finally, no statistically significant association has been observed between *MBL2* genotypes, haplotypes and combined genotypes, and the clinical characteristics of OLP patients. The lack of association might be due to the low number of subjects with OLP after stratification for clinical characteristics. This issue should be reconsidered in a larger group of OLP patients, where the severity of the disease could be categorized, allowing a more robust statistical comparison between *MBL2* genetic variants and the patients’ phenotype.

In conclusion, the association between the X allele, C/T genotype of the D allele, and MBP-C deficient producers combined genotypes, and susceptibility to develop OLP was supported by a quite high statistical power. However, replica studies recruiting larger cohorts of different ethnic origins are necessary to understand the role of *MBL2* genetic variants in the susceptibility of OLP development, bearing in mind that, being a multifactorial disease, other genetic variants are cumulatively involved in OLP etio-pathogenesis.
